# What is the efficacy of aerobic exercise versus strength training in the treatment of migraine? A systematic review and network meta-analysis of clinical trials

**DOI:** 10.1186/s10194-022-01503-y

**Published:** 2022-10-13

**Authors:** Yohannes W. Woldeamanuel, Arão B. D. Oliveira

**Affiliations:** 1 Division of Headache & Facial Pain, Department of Neurology & Neurological Sciences, Stanford University School of Medicine, Stanford, USA; 2 Center for Clinical and Epidemiological Research, Hospital Universitário, Universidade de São Paulo, São Paulo, Brazil

**Keywords:** Aerobic exercise, Exercise, Migraine, Network meta-analysis, Strength training, Systematic review

## Abstract

**Background:**

Multiple clinical trials with different exercise protocols have demonstrated efficacy in the management of migraine. However, there is no head-to-head comparison of efficacy between the different exercise interventions.

**Methods:**

A systematic review and network meta-analysis was performed involving all clinical trials which determined the efficacy of exercise interventions in reducing the frequency of monthly migraine. Medical journal search engines included Web of Science, PubMed, and Scopus spanning all previous years up to July 30, 2022. Both aerobic and strength/resistance training protocols were included. The mean difference (MD, 95% confidence interval) in monthly migraine frequency from baseline to end-of-intervention between the active and control arms was used as an outcome measure. Efficacy evidence from direct and indirect comparisons was combined by conducting a random effects model network meta-analysis. The efficacy of the three exercise protocols was compared, i.e., moderate-intensity aerobic exercise, high-intensity aerobic exercise, and strength/resistance training. Studies that compared the efficacy of migraine medications (topiramate, amitriptyline) to exercise were included. Additionally, the risk of bias in all included studies was assessed by using the Cochrane Risk of Bias version 2 (RoB2).

**Results:**

There were 21 published clinical trials that involved a total of 1195 migraine patients with a mean age of 35 years and a female-to-male ratio of 6.7. There were 27 pairwise comparisons and 8 indirect comparisons. The rank of the interventions was as follows: strength training (MD = -3.55 [− 6.15, − 0.95]), high-intensity aerobic exercise (-3.13 [-5.28, -0.97]), moderate-intensity aerobic exercise (-2.18 [-3.25, -1.11]), topiramate (-0.98 [-4.16, 2.20]), placebo, amitriptyline (3.82 [− 1.03, 8.68]). The RoB2 assessment showed that 85% of the included studies demonstrated low risk of bias, while 15% indicated high risk of bias for intention-to-treat analysis. Sources of high risk of bias include randomization process and handling of missing outcome data.

**Conclusion:**

Strength training exercise regimens demonstrated the highest efficacy in reducing migraine burden, followed by high-intensity aerobic exercise.

**Supplementary Information:**

The online version contains supplementary material available at 10.1186/s10194-022-01503-y.

## Introduction

Migraine is highly prevalent and has a high socio-economic impact [1–4]. The understanding of treatments that can contribute to reduction in migraine burden is therefore of major importance. Exercise, defined as any type of planned, structured, and repetitive movement/physical activity performed to improve and/or maintain one or more components of physical fitness (e.g., aerobic fitness, muscle strength, etc.) [5, 6] provides therapeutic effects on migraine [7–10]. Several clinical trials have demonstrated the efficacy of exercise interventions in the management of migraine [10–16].

However, the exercise interventions involved different types of exercise modalities, i.e., aerobic exercise (high-intensity aerobic exercise, moderate-intensity aerobic exercise), strength training, or even multimodal exercise training (i.e., aerobic, strength, flexibility) protocols [10–17]. Direct head-to-head comparisons between the different exercise protocols are lacking in the literature, e.g., aerobic exercise versus strength training. The variable efficacy results from these different exercise protocols hamper the precise identification of specific treatment effects of each exercise modality. For instance, it is challenging to simultaneously weigh the efficacy results of a clinical trial comparing aerobic exercise to placebo against another clinical trial comparing strength training to placebo. In the absence of such direct evidence, this study aimed to provide indirect comparisons by utilizing a systematic review with a network meta-analysis (NMA). In brief, an NMA, also known as mixed treatment meta-analysis or indirect meta-analysis is an expansion of the conventional pairwise meta-analysis whereby different interventions can be compared. By so doing, NMA allows a coherent approach to not only compare multiple interventions where head-to-head comparisons are lacking, but also to rank the efficacy of the different interventions. Increasing the knowledge of the most efficient treatment can contribute to non-pharmacological migraine-tailored treatments.

## Methods

### Search methods

The search method was conducted by using 3 search engines: Web of Science Advanced Search: Web of Science Core Collection, PubMed Advanced Search Builder, Scopus Advanced Search. The combination of these 3 search engines has been demonstrated to be the most optimum search tool for biomedical literature [18, 19]. For Web of Science Advanced Search, the search method, terms, and Boolean operator were: TS = (migraine* exercise AND clinical trial) and TS = (migraine* exercise). TS stands for topics, while the asterisk * is a wildcard that will find similar items e.g., migrainous, migraineur, etc. For PubMed Advanced Search Builder, “headache AND exercise”, “migraine AND exercise”, “migraine AND exercise AND clinical trial” were used. For Scopus Advanced Search, the following combinations were used: TITLE-ABS-KEY (migraine AND exercise AND clinical trial) and TITLE-ABS-KEY (headache AND exercise AND clinical AND trial). TITLE-ABS-KEY stands for title of an article, abstract of an article, and keywords provided by the article. The search spanned all previous years up to July 30, 2022 for all articles published in English. Articles published in other languages were included if the abstracts with results were available in English. PROSPERO (Prospective Register of Systematic Reviews) acknowledgement of receipt was 354,276.

### Inclusion and exclusion criteria

Controlled clinical trial studies, including studies that utilized randomization, non-randomized arm, and historical control arm, and which involved an exercise regimen as an intervention arm compared to a control arm with no intervention or usual care were included. Additional inclusion criteria were studies that reported monthly frequency of migraine at baseline and at the end of the intervention. Authors of studies that reported the outcomes as a variant of migraine frequency (e.g., 50% frequency reduction) were requested to share baseline and end-of-intervention monthly migraine frequency data. Studies that enrolled adult patients (age 18 years and older) with episodic as well as chronic migraine were included. The exclusion criteria were single-arm studies involving pre-post analysis, cross-sectional studies, case–control studies, retrospective studies, studies with children (younger than age 18 years), and studies that are not clinical trials. PRISMA (Preferred Reporting Items for Systematic Reviews and Meta-Analyses) [20] flowchart was used to depict the identification, screening and inclusion/exclusion of studies.

### Data extraction

The following datapoints were extracted from each included study: first author name, year of publication, interventions, sample size in each arm, mean difference and pooled standard deviation in monthly migraine frequency between baseline and end of intervention, and duration of exercise intervention protocol.

### Risk of bias assessment

Risk of bias assessment of the included studies was conducted using the Risk of Bias version 2 tool (RoB2) [21] for the following domains: randomization process, deviations from intended interventions, missing outcome data, measurement of the outcome, and selection of the reported result.

### Statistical analysis

For descriptive statistics, mean (with standard deviation (SD)) and frequency (with percentage) were used as measures of central tendency and measures of frequency, respectively. A random-effects model on frequentist and Bayesian network meta-analysis was performed to obtain the direct and indirect treatment effect estimates. The random-effects model was preferred over the fixed-effects model as the former allows study variability of the true effect size and can be applied beyond the included studies [22]. Random-effects model recognizes inter-study heterogeneity in terms of methodology and other covariates [22].

Baysesian NMA was performed using the gemtc package on R (https://cran.r-project.org/web/packages/gemtc/index.html). Heterogeneity standard deviation was estimated using the uniform prior U(0,X), where X represents the highest difference in the analysis' outcome scale determined from the data. Bayesian NMA results included the leverage plot for assessing model fitness, per-arm residual deviance for all studies (displayed as a stem plot), and residual deviance from the NMA model and unrelated mean effects (UME) inconsistency model for all included studies. The statistical details of the Bayesian and frequentist NMA are described in detail on the MetaInsight [22] online platform – the software used for this study’s analysis. A sensitivity analysis was performed to examine whether the efficacy results vary between patients with episodic and chronic migraine.

NMA results were visualized using forest plots. Relative treatment effects were ranked in descending order. Assessment of inconsistency for all studies was made to compare the difference between the observed mean differences (direct evidences from pairwise comparisons) and the NMA estimations (indirect evidences). A *p*-value of 0.05 was selected as a level of significant inconsistency. All mean differences were accompanied by a 95% confidence interval. Statistical analysis was performed using the MetaInsight online platform [22].

## Availability of data and materials

All data generated or analyzed during this study are included in this published article and its Supplementary File.

## Results

### Summary of included articles

**Fig. 1:**
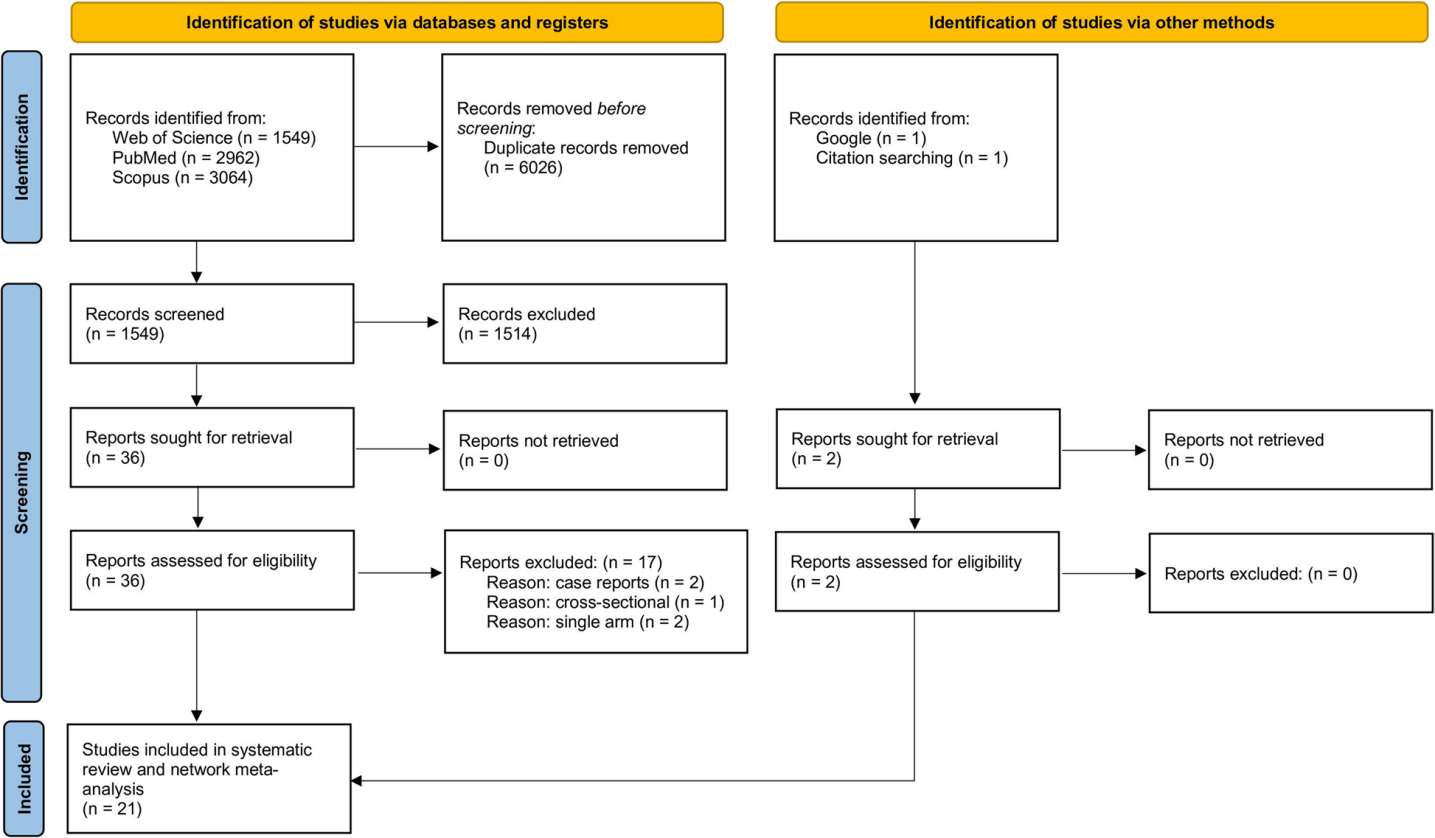
PRISMA (Preferred Reporting of Items in Systematic Reviews and Meta-Analysis) flowchart depicting the identification, screening, and inclusion of the studies included

The number of articles identified and screened for eligibility was as follows. On Web of Science Advanced Search: Web of Science Core Collection TS = (migraine* exercise AND clinical trial) = 180, TS = (migraine* exercise) = 1369. For PubMed Advanced Search Builder, “headache AND exercise” = 1886, “migraine AND exercise” = 614, and “migraine AND exercise AND clinical trial” = 462. For Scopus Advanced Search, TITLE-ABS-KEY (migraine AND exercise AND clinical trial) = 305 and TITLE-ABS-KEY (headache AND exercise AND clinical AND trial) = 2759. The final number of articles included was 21 [10, 12–16, 23–37]. See Fig. 1 for PRISMA [20] flowchart depicting a summary of the identification, screening and inclusion/exclusion of articles.

**Fig. 2:**
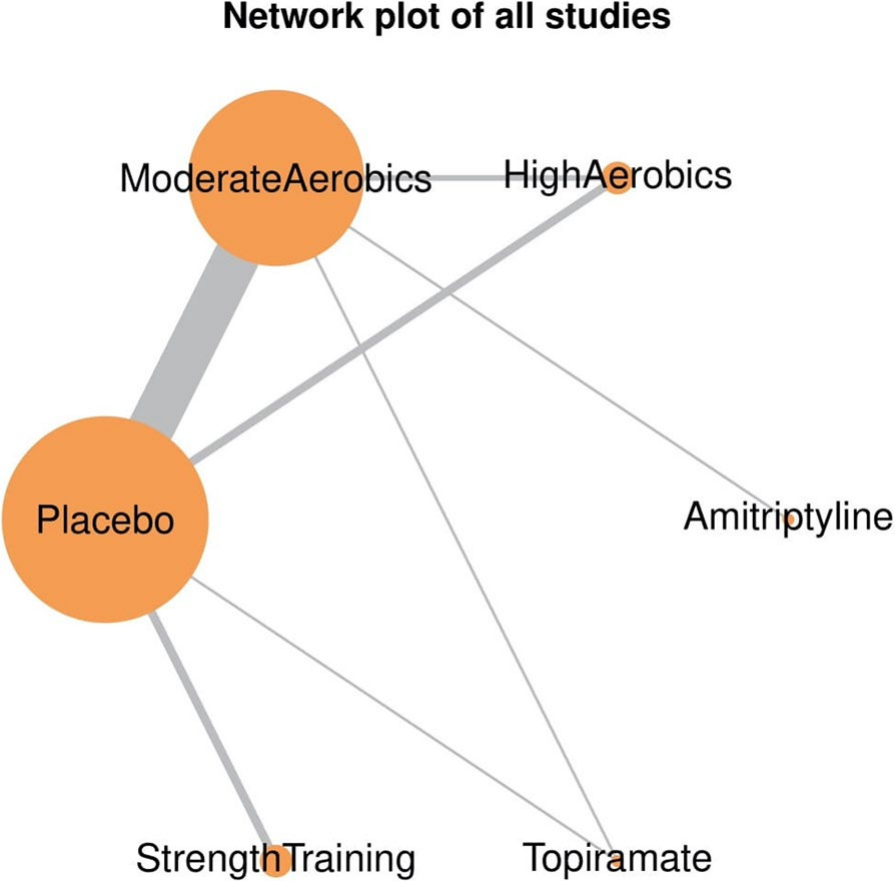
Network plot of all studies included. The size of the nodes and the thickness of edges depend on the number of people randomized and the number of trials conducted, respectively

Of the 21 published clinical trials included in this study, 18 were two-arm and 3 three-arm clinical trials. Eight corresponding authors were requested to provide migraine-specific outcome data i.e., monthly migraine frequency at baseline and end of intervention; 3 authors provided this migraine-specific data while 1 author confirmed that such outcome was not collected in the study. The combined sample size was a total of 1195 migraine patients (mean age of 35.5 years and female-to-male ratio of 6.7:1). All studies used International Classification of Headache Disorders (ICHD) [38] criteria for migraine diagnosis. Of the 21 studies included, 9 (43%) [10, 12, 15, 27, 28, 30, 31, 36, 37] involved patients with chronic migraine out of which 2 studies enrolled exclusively patients with chronic migraine. The NMA provided 27 pairwise comparisons and 8 indirect comparisons. The pairwise head-to-head comparisons provided direct evidence between the different interventions, i.e., placebo *vs* moderate-intensity aerobic exercise, placebo *vs* high-intensity aerobic exercise, placebo *vs* strength training, placebo *vs* topiramate, moderate-intensity aerobic exercise *vs* topiramate, moderate-intensity aerobic exercise *vs* high-intensity aerobic exercise, moderate-intensity aerobic exercise *vs* amitriptyline. Intention-to-treat analysis was used by 71.4% (15) of the included clinical trials, while the remaining 29.6% (6) used per-protocol analysis. A network plot showing the pairwise comparisons is displayed in Fig. 2.

When combining all intervention types, the most common durations of the exercise interventions were 12-week (40%) and 8-week (40%). The average number of weeks for the strength/resistance training, high-intensity aerobic exercise, and moderate-intensity aerobic exercise interventions were 9.3 (SD = 2.3; range = 8 – 12), 9.3 (SD = 2.3; range = 8 – 12), and 10.7 (SD = 4.8; range 6 – 28 weeks), respectively. The average number of hours per session for the strength/resistance training, high-intensity aerobic exercise, and moderate-intensity aerobic exercise interventions were 50 (SD = 14; range = 40 – 60), 56 (SD = 7; range = 48 – 60), and 45.3 (SD = 10; range 30 – 60 weeks), respectively. All workouts involved 10–20 min of warm-up and cool-down periods, while some strength/resistance training workouts included pre- and post-workout stretching.

Strength/resistance training was initiated with 2–3 sets of 12–15 repetitions at a capacity of 45–60% of the maximum weight lifted in a single repetition (one repetition maximum or one RM) [23, 36] performed thrice a week These workouts progressed by 5% one RM each week to reach a target of 75–80% of one RM with 3 sets of 8–10 repetitions by 8–12 weeks [23, 36]. The weight/resistance training included deep flexor and extensor muscles of the cervical region, superficial muscles of the cervical region, shoulder external and internal rotations, seated row, shoulder extension and flexions, press and curl, butterfly and reversed butterfly, leg extension, and latissimus pull down [10, 23, 36]. One strength/resistance training study included music during training sessions to provide a pleasant affect [36].

Some of the aerobic exercise studies utilized advanced aerobic exercise measurements such as a treadmill with breath-by-breath spirometric gas-exchange data, capillary blood samples for lactate measurements taken from the earlobe to determine the participant’s anaerobic lactate-threshold, maximal heart rate (HR_max_), and maximum rate of oxygen consumption during exercise (VO_2max_) [16, 28, 33]. Some of the moderate-intensity aerobic exercise studies used personalized training program by initiating with 45–70% VO_2max_ and 60–80% HR_max_ conducted thrice per week with progressive weekly increments in intensity [16, 33]. The high-intensity aerobic exercise studies involved a high-intensity interval training initiated at a 55–60% VO_2max_ performed 2–3 times a week, raising it weekly by 5–10% VO_2max_ to reach a target of 80–90% VO_2max_ as well as 90–95% HR_max_ target by 8–12 weeks [16, 28, 33]. In one high-intensity aerobic exercise study, the intervals were comprised of a period of 1-min high-intensity aerobic exercise followed by an active recovery aerobic exercise of 70% HR_max_ for a 4-min interval, repeated for 30–60 min, including 10–20 min of warm-up and cool-down periods [16]. The workouts used in both the high-intensity and moderate-intensity aerobic exercise interventions included running outdoors as well as on a treadmill, jump rope, stationary bicycles, and other home-based exercises.

Self-report measures such as the Borg’s rating of perceived exertion (RPE) scale (range 6–20 with high scores indicating high exertion [39]) were used by some of the moderate-intensity aerobic exercise studies to personalize the intensity of the aerobic exercises [14, 31]. The main exercise period for these moderate-intensity aerobic exercise interventions was targeted to reach an RPE of 14–16 while the warm-up and cool-down periods were lowered to an RPE of 11–13 [14, 31, 39].

### Risk of bias assessment

**Fig. 3:**
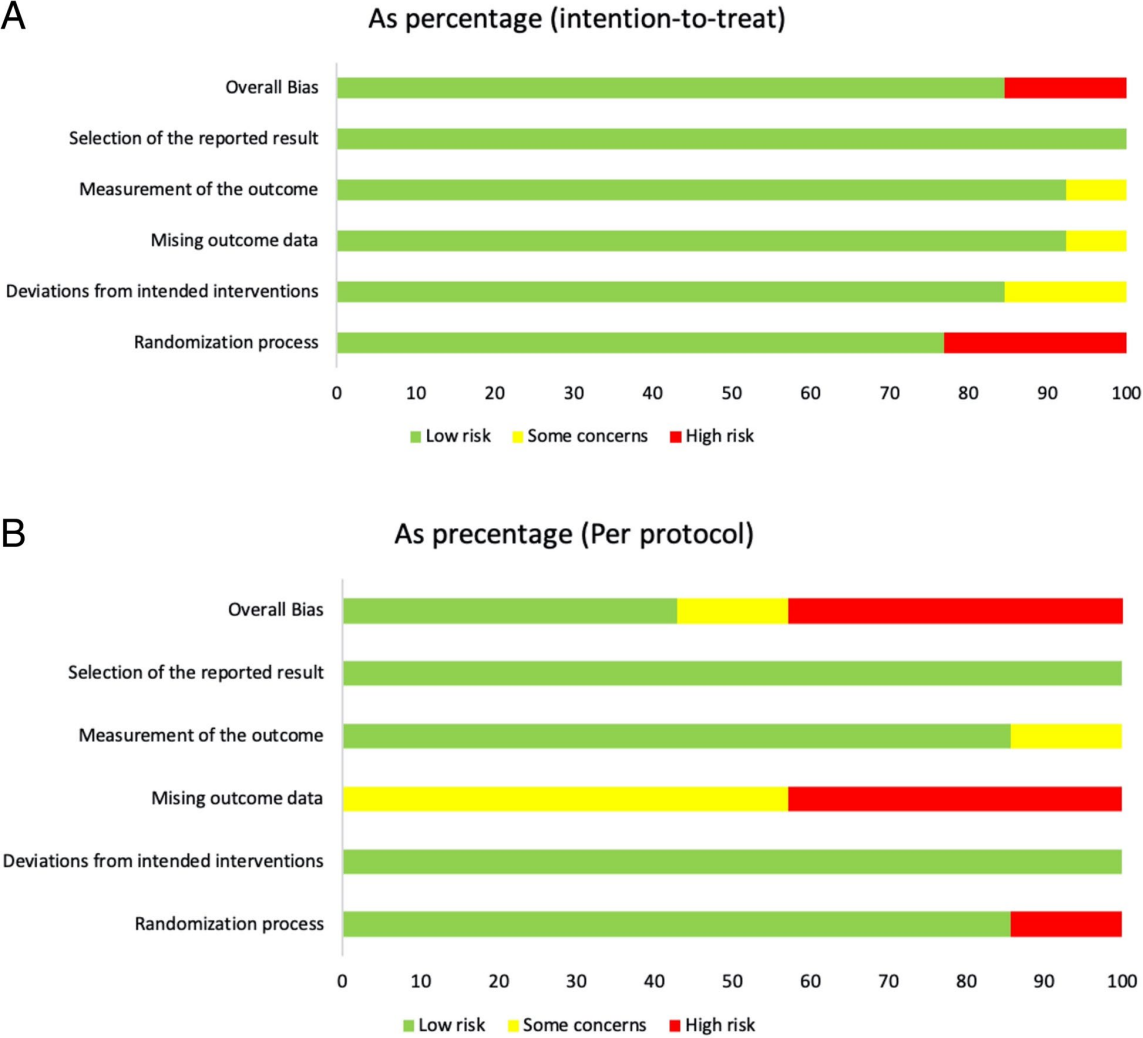
**A** Risk of Bias summary for included studies which applied intention-to-treat analysis. **B** Risk of Bias summary for included studies which applied per-protocol analysis

According to the RoB2 assessment, 85% of the included studies demonstrated low risk of bias, while 15% indicated high risk of bias for intention-to-treat analysis. For studies using per-protocol analysis, 43%, 14%, and 43% of the studies showed low risk of bias, some concerns, and high risk of bias, respectively. Sources of high risk of bias include the randomization process and handling of missing outcome data. Detailed RoB results are displayed in Fig. 3A and B as well as Supplementary Fig. 2 and Supplementary Fig.3.

### Network Meta-analysis (NMA)

**Fig. 4:**
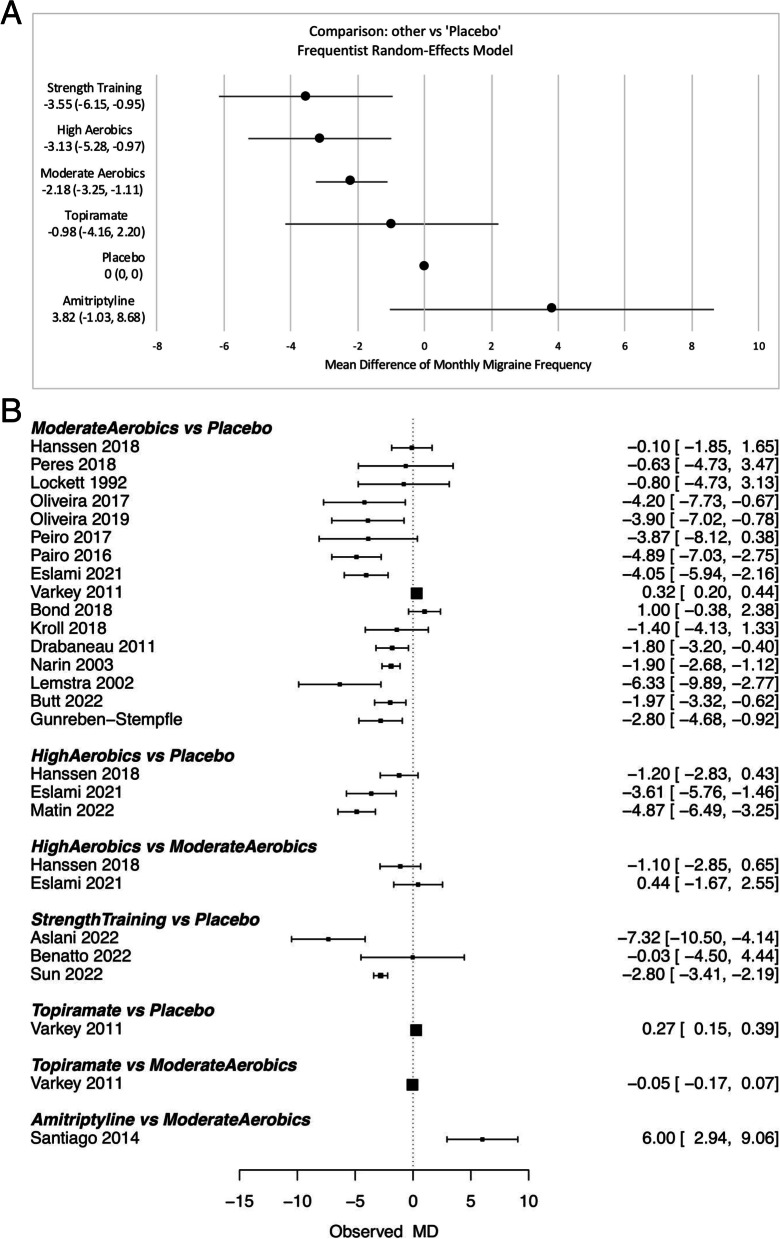
**A** Forest plot for comparison of all interventions against the reference (i.e. placebo) using the frequentist random-effect model network meta-analysis. **B** Individual study results (for all studies) grouped by treatment comparison. Forest plot was based on random-effects model for network meta-analysis. MD = mean difference between active and placebo arms in monthly migraine frequency from baseline to end of intervention

Both the frequentist and Bayesian NMA produced similar results and ranks (Fig. 4A, Supplementary Fig. 1). When the efficacy results from all interventions were compared to placebo using frequentist NMA, the first rank was for strength training with a mean difference in monthly migraine days of -3.55 [− 6.15, − 0.95]), between the active and placebo arms (Fig. 4A, Table 1). The remaining ranks were as follows, in a descending order: high-intensity aerobic exercise (-3.13 [-5.28, -0.97]), moderate-intensity aerobic exercise (-2.18 [-3.25, -1.11]), topiramate (-0.98 [-4.16, 2.20]), placebo, amitriptyline (3.82 [− 1.03, 8.68]) (Fig. 4A, Table 1). Individual study results (for all studies) grouped by treatment comparison are shown in Fig. 4B. There were no statistically significant differences between the direct (pairwise) and indirect (NMA) efficacy estimates in all comparisons (Table 1).

**Table 1:** Assessment of inconsistency between direct and indirect comparisons of interventions. Direct, NMA, and indirect values represent effect sizes of mean difference between active and placebo arms in monthly migraine frequency from baseline to end of intervention. NMA = network meta-analysis

Comparison	Number of Studies	NMA	Direct Comparison	Indirect Comparison	Difference between Direct & Indirect Comparison	*p*-value
StrengthTraining:Placebo	3	-3.55	-3.55	NA	NA	NA
HighAerobics:Placebo	3	-3.13	-3.21	-2.62	-0.60	0.85
StrengthTraining:Topiramate	0	-2.57	NA	-2.57	NA	NA
ModerateAerobics:Placebo	16	-2.18	-2.11	-7.08	4.96	0.31
HighAerobics:Topiramate	0	-2.15	NA	-2.15	NA	NA
ModerateAerobics:Topiramate	1	-1.20	0.05	-5.42	5.47	0.16
Topiramate:Placebo	1	-0.98	0.27	-5.20	5.47	0.16
HighAerobics:ModerateAerobics	2	-0.95	-0.36	-1.80	1.44	0.53
HighAerobics:StrengthTraining	0	0.42	NA	0.42	NA	NA
ModerateAerobics:StrengthTraining	0	1.37	NA	1.37	NA	NA
Amitriptyline:Placebo	0	3.82	NA	3.82	NA	NA
Amitriptyline:Topiramate	0	4.80	NA	4.80	NA	NA
Amitriptyline:ModerateAerobics	1	6	6	NA	NA	NA
Amitriptyline:HighAerobics	0	6.95	NA	6.95	NA	NA
Amitriptyline:StrengthTraining	0	7.37	NA	7.37	NA	NA

*NA* not available, *NMA* network meta-analysis

**Fig. 5:**
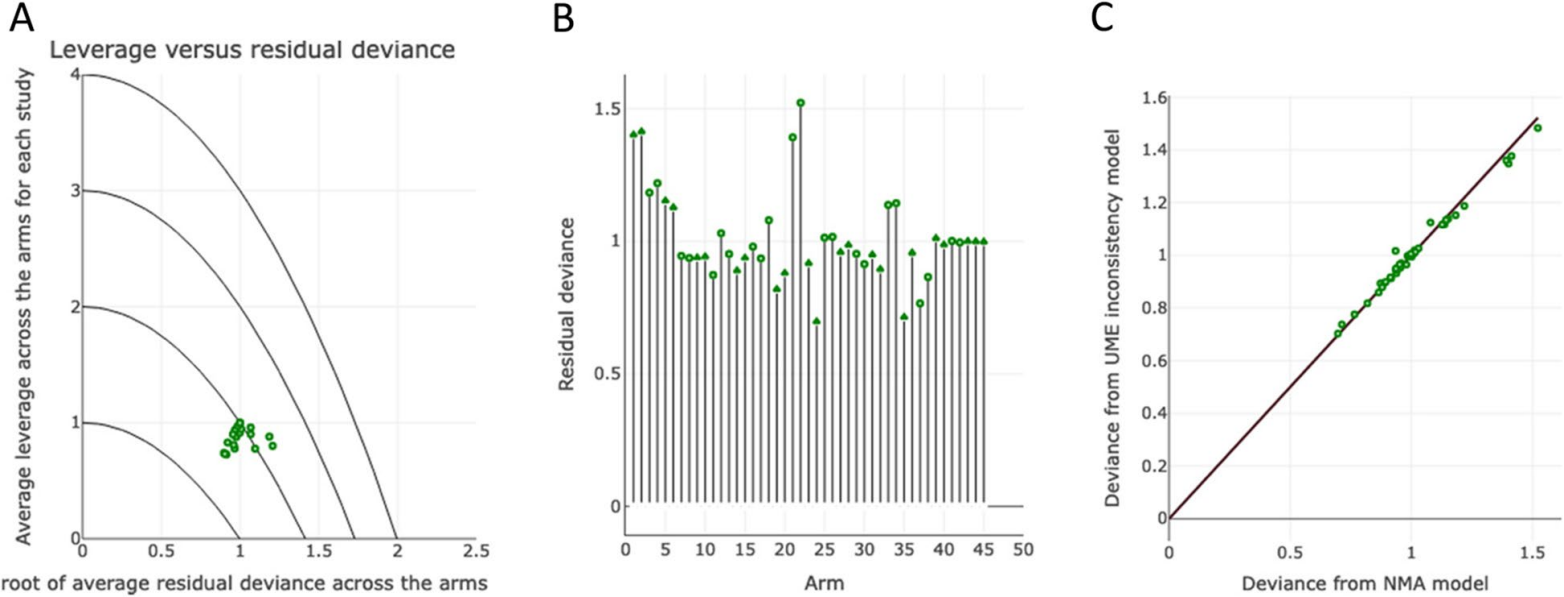
**A** Leverage *versus* residual deviance. The leverage plot helps to assess model fitness. Points that lie outside the line with average leverage of 3 can generally be identified as contributing to the model's poor fit. All included studies (represented by the green dots) were congregated between the average leverage parabola lines of 1.5 and 2.5, indicating that all studies fitted well in the model. **B** The stem plot represents the posterior residual deviance of each study arm. The number of stems represents the 43 study arms compared in the network meta-analysis. Each stem indicates the residual deviance of each arm in each included study. The shorter the stem, the smaller the residual deviance, the better the model fit for each data point. None of the stems featured residual deviance greater than 2 – reflecting the model’s fitness. **C** This plot represents each arm’s contribution to the residual deviance for the NMA (x-axis) and the unrelated mean effect (UME) inconsistency models (y-axis) along with the line of equality. The points on the equality line means there is no improvement in model fit when using the inconsistency model, suggesting that there is no evidence of inconsistency. Points above the equality line means they have a smaller residual deviance for the consistency model indicating a better fit in the NMA consistency model and points below the equality line means they have a better fit in the UME inconsistency model. Most of the included study arms were found on the equality line indicating minimal inconsistency and optimum model fitness

The leverage plot for assessing model fitness demonstrated that all included studies (green dots in Fig. 5A) were assembled between the average leverage parabola lines of 1.5 and 2.5 – indicating that all studies fitted well in the model. The stem plot (Fig. 5B) displayed the residual deviance of each of the 43 study arms in the network meta-analysis. The shorter the stem, the smaller the residual deviance, and the better the model fitness for each study arm. All the stems featured residual deviance lower than 2 – reflecting the model’s fitness. The plot for residual deviance from the NMA model and UME inconsistency model showed that most of the included study arms (green dots in Fig. 5C) congregated along the equality line, indicating minimal inconsistency and optimal model fitness. Sensitivity analysis was not undertaken, since the NMA models showed adequate goodness-of-fit measurements. The two studies with moderate-intensity aerobic exercise exclusively involving chronic migraine patients showed a large effect size (Cohen’s *d*) of 0.80 and 1.10 in reducing monthly headache frequency.

## Discussion

This systematic review and NMA showed that while all exercise intervention protocols are more efficacious than the placebo arms in reducing the frequency of migraine, strength/resistance training resulted in the highest efficacy followed by high-intensity and moderate-intensity aerobic exercise. The summary in risk of bias assessment was low risk for the majority of the studies – indicating the validity of the meta-evidence in estimating the true treatment effects.

The reason for strength/resistance training to rank top could be because of targeted muscular strengthening and reconditioning, particularly involving major muscles in the neck, shoulders and upper limbs. All exercise modalities showed higher therapeutic effects compared to placebo. Neck pain is highly comorbid with migraine in clinical populations [40–43]. The nociceptive input from neck structures of the upper cervical spine is connected with the trigemino-cervical system, the neuroanatomic spot of migraine disorders [44, 45]. The nociceptive input into the spinal cord can be modulated by segmental mechanisms in the spinal cord and by inhibitory projections from brain stem structures (e.g., the periaqueductal gray) [44, 45]. Local metabolic and neuromuscular adaptations and the related strength increase induced by neck strength exercise could be putative mechanisms underlying the therapeutic effects of neck exercises.

Regarding aerobic exercise, the superior effects of high *versus* moderate-intensity aerobic exercise may be linked to intensity-specific recruitment of endogenous molecules involved in exercise-mediated hypoalgesia. Exercise-mediated hypoalgesia involves both opioid and endocannabinoid systems [46–51]. Migraine has been linked to a deficiency of both opioidergic [52] and endocannabinoidergic [53] signaling, and both are a target of current translational migraine models [54–56]. Interestingly, evidence from human studies shows that peripheral endorphin secretion and brain opioid receptor binding occur preferably during high-intensity rather than moderate aerobic exercise [46, 49, 50], while endocannabinoids (e.g., anandamide, 2-arachidonoyl-glycerol) are released preferably during moderate, rather than high-intensity aerobic exercise [13, 57]. However, the studies assessing the opioid/endocannabinoid systems and exercise have investigated majorly the acute response (i.e., a single exercise session) to exercise [46–51]. In people with migraine, only two studies – one for circulating endorphins [58], the other for circulating anandamide – investigated the long-term response (aerobic training effect) to moderate aerobic exercise [13]. There was no change in the post-intervention circulating β-endorphin levels or associations between the changes in the circulating β-endorphin levels and migraine clinical outcomes, while anandamide was reduced after the intervention period and this change was significantly correlated with lower abortive medication use [13]. The interpretation of these results is that circulating β-endorphin was not adequate to reflect the change in the central nervous system opioidergic signaling and/or moderate aerobic exercise is not sufficient to stimulate this system. Regarding anandamide, a decrease in circulating levels may reflect peripheral rather than central metabolism, which may have implications for exercise-induced anti-obesogenic/diabetogenic processes, or for the dual pronociceptive action of anandamide via activation of transient receptor potential vanilloid type 1 (TRPV1) receptors [13]. To date, there is no study with migraine patients investigating high-intensity aerobic exercise and the opioid/endocannabinoid systems. Thus, the intensity-specific clinical benefit of aerobic exercise in migraine could be influenced by patients’ endogenous opioidergic/endocannabinoidergic profile. Furthermore, the multifactorial pathophysiology of migraine may involve abnormal musculoskeletal/brain energy metabolism and mitochondrial disfunction [59], and inflammation [60, 61]. This apparent dose-dependent effect of aerobic exercise on migraine could be mediated by improved mitochondrial/cardiorespiratory function [17], as well as by anti-inflammatory mechanisms associated with aerobic exercise [26, 62].

Another reason why strength/resistance training outperformed aerobics exercise in reducing migraine burden could be related to its higher capacity to increase/preserve lean muscle mass and combat sarcopenia (while still losing fat) compared to aerobics [63]. Increased lean muscle mass is known to be associated with reduced migraine frequency [64, 65]. Moreover, increment/preservation of lean body mass has been shown to combat central sensitization in chronic pain syndromes [66]. This indicates that the type of weight loss determines the efficacy of exercise interventions. Weight loss involving fat loss as seen in strength/resistance training is different than weight loss associated with muscle loss as seen in aerobic training [63].

Based on our meta-analysis and experience, we can infer that combining strength/resistance training days and active recovery days may provide the most optimum efficacy in reducing migraine burden. Additionally, utilizing such combinations on different days provides the necessary gap for recovery time between the various major muscle groups exercised. Active recovery exercise involves individualized low-intensity exercise [67]. An easy and validated tool to measure exercise intensity is by using the talk test [68]. In brief, during an active recovery exercise, a person can hold steady conversations while doing the exercise [68]. Multiple studies have shown that active recovery is better than passive recovery (e.g., complete rest) in reducing exercise-related fatigue, delayed-onset muscle soreness, inflammation, and muscle damage [67, 69].

For strength/resistance training, based on this study’s findings, our recommendations are to start with 50% one RM with 2–3 sets of 12–15 repetitions done thrice a week along with 10 min of warm-up, stretching, and cool-down totaling 45–60 min per session. Subsequently, weight/resistance load can be increased weekly by 5% one RM if the patient is capable of successfully completing 3 sets. We also recommend including active recovery days (low-intensity exercise) in between training days. All major muscles, including neck muscles, need to be trained in a rotation, e.g., day-1: neck, shoulders, upper limbs; day-2: active recovery; day-3: glutes, thighs, calf muscles; day-4: active recovery; day-5: core and back muscles; day-6: active recovery; day-7: repeat day-1.

For high-intensity aerobic exercise, our recommendations, based on this study’s findings, are to start with high-intensity interval training at 55% VO_2max_ or 50% HR_max_ (RPE = 14–16; talk test = able to hold a conversation) for 45–60 min per session including 10 min of warm-up and cool-down, performed thrice per week. Subsequently, the intensity can be increased by 5–10% each week depending on the patient’s capacity to reach a target of 80–90% VO_2max_ as well as 90–95% HR_max_ (RPE = 17–20; talk test = only able to say 1–2 words between breathing) by 8–12 weeks. Our recommendations are to perform a 1-min high-intensity followed by 4-min moderate-intensity aerobic exercise. For moderate-intensity, our recommendation is 50–55% VO_2max_ or 50–60% HR_max_ (RPE = 14–16; talk test = able to hold a conversation). Including music in the background may also enhance the performance of the workout sessions.

From a practical viewpoint, the results from this NMA study and the following exercise prescription proposed here for migraine populations are in line with the current WHO physical activity guidelines [70]. The current World Health Organization´s (WHO) physical activity guidelines for health promotion in adults recommend a weekly amount of at least 150 min of moderate aerobic physical activity and/or 75 min of high (vigorous) aerobic physical activity or an equivalent combination of moderate/vigorous physical activity. Additionally, the WHO guidelines recommend adding full-body resistance training (muscle-strengthening activities) at moderate or greater intensity 2 or more days a week [70]. Agreeably, recent cross-sectional cohort studies have shown that people that meet the WHO physical activity guidelines have lower odds of migraine [71, 72].

Based on the duration (median of 50 min based on this study) and frequency (3 times a week based on this study) of exercise sessions adopted in the studies included in this NMA, combining strength/resistance training and aerobic exercise within an exercise training framework that meets WHO´s guidelines, that is 150 min/week of moderate aerobic exercise or 75 min/week vigorous aerobic exercise (or combination of both) and strength/resistance training 2 times a week, allows migraine care specialists to tailor a variety of exercise using different modality and intensity on different days that better fit patients’ reality and context, and to provide adequate recovery time from exercise sessions with higher intensities. Thus, a multimodal exercise training program may provide the most optimum efficacy in reducing migraine burden. Other factors to be considered while tailoring an exercise program for people with migraine are the training load progression, initial fitness status, and personal preferences (aerobic *vs* resistance).

A regular exercise intervention that incorporates personalized and graded exercise exposure can improve the therapeutic benefits of exercise in migraine. We recommend that migraine patients need to be advised that some level of headache flare-up is normal with physical activity. Exercise-related migraine flare-ups need to be regarded as a protective strategy and not necessarily a sign of new damage. In our experience, the best way to manage exercise-related migraine flare-ups is by using pacing strategies to find the middle-of-the-road – continuing manageable physical activity, and not stopping. Here, regularity is the key, not necessarily the volume or intensity of exercise. A pacing strategy, in brief, involves not going overboard on good days and avoiding excessive rest on bad days. During periods of high scalp cutaneous sensitivity, exercising non-painful parts of the body can be an option.

The limitations of this study include the imbalances in the number of studies comparing the different exercise interventions: as such, results from a comparison with fewer studies may not have been accurately appraised. In addition, some of the included studies [30, 32, 35] embedded exercise protocols within a multi-component intervention (e.g., eating behavior change along with exercise intervention [37]). As a consequence, effect size estimations for the different exercise interventions may not be precise. Neck strength exercises and whole-body resistance training were analyzed together under strength/resistance training [10, 23, 30, 36]. The NMA accepts clinical outcomes that were not originally designed as primary outcomes of randomized controlled trials. Nonetheless, an increase in the overall study power is generally realized by utilizing a systematic review and meta-analysis. Some of the included studies did not have a sample size estimation a priori. Excluded studies lacked monthly migraine frequency as an outcome of their intervention. Monthly migraine frequency is the recommended outcome measurand for migraine clinical trials [73, 74]. Sensitivity analysis should be included in the studies reporting their results using the per-protocol approach, so as to ensure missing data outcome was not a source of bias for effect sizes. As per our risk of bias assessment, information on missing data handling techniques and details on the randomization process need to be addressed in future studies.

Personalizing exercise modalities as per the participant’s preference enhances its efficacy. Regular exercising can have a lasting impact in controlling migraine through a healthy lifestyle behavior change. A dedicated leisure-time physical activity (e.g., gym training, weightlifting, individual or group sport) is associated with lower migraine burden compared to physical activity during commuting or doing errands [72]. By virtue of being a lifestyle-based intervention, regular exercise not only helps reduce migraine attacks, but also helps control other known comorbidities such as obesity, depression, and insomnia.

## Supplementary Information

**Additional file 1:** **Supplementary Table 1.** Dataset extracted from each included study and analyzed in the network meta-analysis. Studies are represented by their first author last name and year of publication. **Supplementary Figure 1.** Forest plot for comparison of all interventions against the reference (i.e. placebo) using the Bayesian random-effect model network meta-analysis. Similar to the forest plot shown in Figure 4A using frequentist NMA, strength/resistance training ranked top in efficacy of reducing migraine frequency – closely followed by high-intensity and moderate-intensity aerobic exercise. **Supplementary Figure 2.** Risk of bias graph showing a review of authors’ judgements on each risk of bias item presented as percentage across all included studies utilizing intention-to-treat analysis. **Supplementary Figure 3.** Risk of bias graph displaying a review of authors’ judgements on each risk of bias item presented as percentage across all included studies utilizing per-protocol analysis.
