# Supplementary material for: What is the efficacy of aerobic exercise versus strength training in the treatment of migraine? A systematic review and network meta-analysis of clinical trials
Source: J Headache Pain. 2022 Oct 13;23(1):134. doi: 10.1186/s10194-022-01503-y (PMC9563744; doi:10.1186/s10194-022-01503-y)
Supplement: Supplementary file 1 — Additional file 1: Supplementary Table 1. Dataset extracted from each included study and analyzed in the network meta-analysis. Studies are represented by their first author last name and year of publication. Supplementary Figure 1. Forest plot for comparison of all interventions against the reference (i.e. placebo) using the Bayesian random-effect model network meta-analysis. Similar to the forest plot shown in Figure 4A using frequentist NMA, strength/resistance training ranked top in efficacy of reducing migraine frequency – closely followed by high-intensity and moderate-intensity aerobic exercise. Supplementary Figure 2. Risk of bias graph showing a review of authors’ judgements on each risk of bias item presented as percentage across all included studies utilizing intention-to-treat analysis. Supplementary Figure 3. Risk of bias graph displaying a review of authors’ judgements on each risk of bias item presented as percentage across all included studies utilizing per-protocol analysis. [file 10194_2022_1503_MOESM1_ESM.docx]

| **Study ID** | **Study** | **Intervention** | **Sample Size** | **Mean** | **Standard Deviation** |
| --- | --- | --- | --- | --- | --- |
| **1** | Hanssen 2018 | Placebo | 12 | -1.2 | 2.03 |
| **1** | Hanssen 2018 | Moderate Aerobics | 15 | -1.3 | 2.6 |
| **1** | Hanssen 2018 | High Aerobics | 15 | -2.4 | 2.29 |
| **2** | Peres 2018 | Placebo | 25 | -11.09 | 6.4 |
| **2** | Peres 2018 | Moderate Aerobics | 25 | -11.72 | 8.26 |
| **3** | Lockett 1992 | Placebo | 9 | -1.2 | 5.34 |
| **3** | Lockett 1992 | Moderate Aerobics | 11 | -2 | 3.08 |
| **4** | Oliveira 2017 | Placebo | 10 | 0.4 | 4.57 |
| **4** | Oliveira 2017 | Moderate Aerobics | 10 | -3.8 | 3.41 |
| **5** | Oliveira 2019 | Placebo | 12 | 0.6 | 4.56 |
| **5** | Oliveira 2019 | Moderate Aerobics | 13 | -3.3 | 3.24 |
| **6** | Peiro 2017 | Placebo | 9 | -1.22 | 2.39 |
| **6** | Peiro 2017 | Moderate Aerobics | 9 | -5.09 | 6.05 |
| **7** | Pairo 2016 | Placebo | 9 | -1.22 | 2.39 |
| **7** | Pairo 2016 | Moderate Aerobics | 10 | -6.11 | 2.35 |
| **8** | Eslami 2021 | Placebo | 15 | -1.22 | 2.7 |
| **8** | Eslami 2021 | Moderate Aerobics | 13 | -5.27 | 2.4 |
| **8** | Eslami 2021 | High Aerobics | 12 | -4.83 | 2.93 |
| **9** | Aslani 2022 | Placebo | 8 | 1.12 | 3.2 |
| **9** | Aslani 2022 | Strength Training | 10 | -6.2 | 3.68 |
| **10** | Santiago 2014 | Amitriptyline | 26 | -12 | 6.36 |
| **10** | Santiago 2014 | Moderate Aerobics | 24 | -18 | 4.6 |
| **11** | Varkey 2011 | Placebo | 30 | -0.97 | 0.23 |
| **11** | Varkey 2011 | Moderate Aerobics | 31 | -0.65 | 0.23 |
| **11** | Varkey 2011 | Topiramate | 30 | -0.7 | 0.23 |
| **12** | Bond 2018 | Placebo | 56 | -4 | 3.56 |
| **12** | Bond 2018 | Moderate Aerobics | 54 | -3 | 3.81 |
| **13** | Kroll 2018 | Placebo | 26 | -0.6 | 4.47 |
| **13** | Kroll 2018 | Moderate Aerobics | 26 | -2 | 5.51 |
| **14** | Drabaneanu 2011 | Placebo | 8 | 0.3 | 1.35 |
| **14** | Drabaneanu 2011 | Moderate Aerobics | 8 | -1.5 | 1.51 |
| **15** | Narin 2003 | Placebo | 20 | -1.9 | 0.85 |
| **15** | Narin 2003 | Moderate Aerobics | 20 | -3.8 | 1.56 |
| **16** | Benatto 2022 | Placebo | 21 | 1.16 | 7.15 |
| **16** | Benatto 2022 | Strength Training | 17 | 1.13 | 6.85 |
| **17** | Lemstra 2002 | Placebo | 36 | -0.47 | 8.24 |
| **17** | Lemstra 2002 | Moderate Aerobics | 44 | -6.8 | 7.86 |
| **18** | Sun 2022 | Placebo | 141 | -4.4 | 2.8 |
| **18** | Sun 2022 | Strength Training | 145 | -7.2 | 2.4 |
| **19** | Butt 2022 | Placebo | 14 | -1.23 | 1.91 |
| **19** | Butt 2022 | Moderate Aerobics | 14 | -3.2 | 1.74 |
| **20** | Gunreben-Stempfle 2009 | Placebo | 80 | -1.3 | 5.01 |
| **20** | Gunreben-Stempfle 2009 | Moderate Aerobics | 42 | -4.1 | 5.04 |
| **21** | Matin 2022 | Placebo | 15 | 0.99 | 2.20 |
| **21** | Matin 2022 | High Aerobics | 15 | -3.88 | 2.32 |

**Supplementary Table 1.** Dataset extracted from each included study and analyzed in the network meta-analysis. Studies are represented by their first author last name and year of publication.

**Supplementary Figure 1.** Forest plot for comparison of all interventions against the reference (i.e. placebo) using the Bayesian random-effect model network meta-analysis. Similar to the forest plot shown in Figure 4A using frequentist NMA, strength/resistance training ranked top in efficacy of reducing migraine frequency – closely followed by high-intensity and moderate-intensity aerobic exercise.

|  | **D1** | **D2** | **D3** | **D4** | **D5** | **Overall** |  |  |  |
| --- | --- | --- | --- | --- | --- | --- | --- | --- | --- |
| Peres 2019 |  |  |  |  |  |  |  |  | Low risk |
| Lockett 1992 |  |  |  |  |  |  |  |  | Some concerns |
| Oliveira 2017 |  |  |  |  |  |  |  |  | High risk |
| Oliveira 2019 |  |  |  |  |  |  |  |  |  |
| Pairo 2016 |  |  |  |  |  |  |  | D1 | Randomisation process |
| Peiro 2017 |  |  |  |  |  |  |  | D2 | Deviations from the intended interventions |
| Bond 2018 |  |  |  |  |  |  |  | D3 | Missing outcome data |
| Kroll 2018 |  |  |  |  |  |  |  | D4 | Measurement of the outcome |
| Narin 2003 |  |  |  |  |  |  |  | D5 | Selection of the reported result |
| Lemstra 2002 |  |  |  |  |  |  |  |  |  |
| Butt 2022 |  |  |  |  |  |  |  |  |  |
| Gunreben-Stempfle 2009 |  |  |  |  |  |  |  |  |  |
| Varkey 20111 |  |  |  |  |  |  |  |  |  |
| Matin 2022 |  |  |  |  |  |  |  |  |  |

**Supplementary Figure 2**. Risk of bias graph showing a review of authors’ judgements on each risk of bias item presented as percentage across all included studies utilizing intention-to-treat analysis.

|  | **D1** | **D2** | **D3** | **D4** | **D5** | **Overall** |  |  |  |  |  |
| --- | --- | --- | --- | --- | --- | --- | --- | --- | --- | --- | --- |
| Hanssen 2018 |  |  |  |  |  |  |  |  | Low risk |  |  |
| Eslami 2021 |  |  |  |  |  |  |  |  | Some concerns |  |  |
| Aslani 2022 |  |  |  |  |  |  |  |  | High risk |  |  |
| Santiago 2014 |  |  |  |  |  |  |  |  |  |  |  |
| Darabaneanu2011 |  |  |  |  |  |  |  | D1 | Randomisation process |  |  |
| Benatto 2022 |  |  |  |  |  |  |  | D2 | Deviations from the intended interventions |  |  |
| Sun 2022 |  |  |  |  |  |  |  | D3 | Missing outcome data |  |  |
|  |  |  |  |  |  |  |  | D4 | Measurement of the outcome |  |  |
|  |  |  |  |  |  |  |  | D5 | Selection of the reported result |  |  |

**Supplementary Figure 3.** Risk of bias graph displaying a review of authors’ judgements on each risk of bias item presented as percentage across all included studies utilizing per-protocol analysis.
